# Fractalkine shedding is mediated by p38 and the ADAM10 protease under pro-inflammatory conditions in human astrocytes

**DOI:** 10.1186/s12974-016-0659-7

**Published:** 2016-08-22

**Authors:** Sinead A. O’Sullivan, Fabrizio Gasparini, Anis K. Mir, Kumlesh K. Dev

**Affiliations:** 1 Drug Development, School of Medicine, Trinity College Dublin, Dublin, Ireland; 2 Novartis Institutes for BioMedical Research, Novartis Pharma AG, Basel, Switzerland

**Keywords:** CX3CL1, Proteolytic processing, Metalloprotease, IL-1β, TNF-α, IFN-γ, NF-kB

## Abstract

**Background:**

The fractalkine (CX3CR1) ligand is expressed in astrocytes and reported to be neuroprotective. When cleaved from the membrane, soluble fractalkine (sCX3CL1) activates the receptor CX3CR1. Although somewhat controversial, CX3CR1 is reported to be expressed in neurons and microglia. The membrane-bound form of CX3CL1 additionally acts as an adhesion molecule for microglia and infiltrating white blood cells. Much research has been done on the role of fractalkine in neuronal cells; however, little is known about the regulation of the CX3CL1 ligand in astrocytes.

**Methods:**

The mechanisms involved in the up-regulation and cleavage of CX3CL1 from human astrocytes were investigated using immunocytochemistry, Q-PCR and ELISA. All statistical analysis was performed using GraphPad Prism 5.

**Results:**

A combination of ADAM17 (TACE) and ADAM10 protease inhibitors was found to attenuate IL-1β-, TNF-α- and IFN-γ-induced sCX3CL1 levels in astrocytes. A specific ADAM10 (but not ADAM17) inhibitor also attenuated these effects, suggesting ADAM10 proteases induce release of sCX3CL1 from stimulated human astrocytes. A p38 MAPK inhibitor also attenuated the levels of sCX3CL1 upon treatment with IL-1β, TNF-α or IFN-γ. In addition, an IKKβ inhibitor significantly reduced the levels of sCX3CL1 induced by IL-1β or TNF-α in a concentration-dependent manner, suggesting a role for the NF-kB pathway.

**Conclusions:**

In conclusion, this study shows that the release of soluble astrocytic fractalkine is regulated by ADAM10 proteases with p38 MAPK also playing a role in the fractalkine shedding event. These findings are important for understanding the role of CX3CL1 in healthy and stimulated astrocytes and may benefit our understanding of this pathway in neuro-inflammatory and neurodegenerative diseases.

**Electronic supplementary material:**

The online version of this article (doi:10.1186/s12974-016-0659-7) contains supplementary material, which is available to authorized users.

## Background

The chemokine fractalkine (CX3CL1) is expressed in the central nervous system (CNS) at relatively high levels in comparison to most other cytokines [1]. CX3CL1 is a transmembrane protein with a chemokine domain attached to a mucin-like stalk [2]. Although somewhat controversial, over the years, it has been shown that the CX3CL1 chemokine is expressed primarily on neurons and astrocytes, with its receptor, CX3CR1, expressed on microglia and neurons [1, 3–8]. Therefore, neurons and astrocytes can signal to neurons and microglia expressing CX3CR1. CX3CL1 can exist in two different forms with the membrane-bound form important for adhesion [9]. CX3CL1 can also be cleaved by ADAM10, ADAM17 or cathepsin S, depending on the cell type and the microenvironment [10, 11]. This cleavage from the cell surface of neurons or astrocytes mainly represents a response to insult or injury. Within the CNS, CX3CL1 has potential to be both neuroprotective and toxic [7, 12–15]. The exact roles for either form of CX3CL1 within the CNS are not completely understood, but in general, because of the high levels in the adult brain, CX3CL1 is thought to help maintain a homeostatic environment as well as respond to CNS insults [4, 5, 16]. Studies also suggest that tonic signalling of the CX3CL1 ligand has an anti-inflammatory effect by somewhat controlling microglial activation states. Therefore, inhibition of this tonic signalling may support inflammatory processes [17].

Transmembrane proteins, such as CX3CL1, can be proteolytically cleaved at the juxtamembrane region, which results in the detachment of their extracellular region (ectodomain) in a process known as ectodomain shedding [18]. This shedding process can release proteins such as cytokines and growth factors from their membrane-bound form, or alternatively, it can down-regulate receptors from the cell surface [18]. Proteases such as the matrix metalloprotease (MMP) family shed several cell surface substrates [19, 20]. One such family of metalloproteases that mediate these events are known as ADAMs (a disintigrin and metalloprotease), of which the tumour necrosis factor alpha (TNF-α)-converting enzyme (TACE/ADAM17) and ADAM10 are most studied [21, 22]. Much work has been done on ADAMs in the periphery, as they are highly abundant in many cell types [11]. Previous studies suggest ADAM17 as the primary protease responsible for inducible cleavage of fractalkine in neurons, with ADAM10 playing a role in constitutive CX3CL1 cleavage [12, 23, 24]. Recent findings suggest that altered function of ADAM10 could contribute to neurodegenerative disease processes such as Alzheimer’s disease and encephalopathies [25, 26]. Importantly, this shedding process of CX3CL1 can be activated by cytokines and growth factors via mitogen-activated protein kinase (MAPK) pathways that involve, among others, p38 signalling [27–30].

Widespread ADAM expression has been demonstrated in the CNS, where a number of previous studies have suggested the expression of ADAM10 and ADAM17 in astrocytes [31]. For example, immunolabelling has revealed increased expression of ADAM10 and ADAM17 associated with reactive astrocytes in murine dentate gyrus [32]. ADAM10 has also been found to be increased in immunohistochemical studies of HIV encephalitis clinical samples in both astrocytes and neurons [33]. Carnosic acid treatment is reported to enhance the messenger RNA (mRNA) expression of ADAM17 in human astrocytoma cells [34], and IL-1α stimulates ADAM17 synthesis in human primary astrocytes [27]. Interleukin-1 beta (IL-1β), TNF-α and interferon gamma (IFN-γ) are archetypical pro-inflammatory mediators, and studies have also reported the expression and activation of receptors for IL-1β [35, 36] and TNF-α on human astrocytes [37]. Human astrocytes have also been shown to produce Aβ40 and Aβ42 when stimulated with IFN-γ and TNF-α or IL-1β [38]. In addition, human and murine astrocytes treated with IFN-γ induced CXCL10 [39]. These cytokines have also been previously shown to increase fractalkine expression on astrocytes [6, 29, 40]. Others and we have also shown previously that human astrocytes respond to these cytokines [41]. We note also that IL-1β and TNF-α signal primarily via nuclear factor kappa b (NF-kB) [37, 42, 43] while IFN-γ signals largely through JAK/STAT [44, 45] allowing for the investigation of two differential pathways.

Many studies performed on peripheral cells implicate ADAM10 in the constitutive shedding of the CX3CL1 ligand from various cell types, while ADAM17 is thought to be the main protease regulating inducible cleavage of this ligand [23]. While a low level of CX3CL1 expression in astrocytes has been reported [3], little information currently exists regarding its regulation, constitutive shedding or inducible cleavage in this cell type. Here, we present new data examining cytokine (IL-1β, TNF-α and IFN-γ)-induced regulation of the CX3CL1 ligand in human astrocytes.

## Methods

### Compounds and cytokines

Inhibitors used ADAM10/ADAM17 inhibitor (BMS-561392), specific ADAM17 inhibitor (BMS-566394), specific ADAM10 inhibitor (TOCRIS; GI 254023X), MMP inhibitor (Millipore; 444289), IKKβ inhibitor (TOCRIS; 2559) and the p38 MAPK inhibitor VX-702 (TOCRIS; 3916) were prepared as 10 mM stock solutions dissolved in 90 % dimethyl sulfoxide (DMSO, Sigma; D8418). The cytokines used were interleukin-1β (IL-1β; R&D Systems, PHC0815), tumour necrosis factor α (TNF-α; R&D Systems, 210-TA) and interferon-γ (IFN-γ; GIBCO, PHC4031).

### Human astrocyte cell culture

Human astrocytes from fetal brains were purchased from ScienCell Research Laboratory, USA (1800, Lot Nos. 9063 and 11065), as per the ethics outlined by the supplier and as we have described previously [41, 46–48]. Human astrocytes were cultured at 37 °C and 5 % CO_2_ in a humidified incubator and grown in human astrocyte speciality media (ScienCell; 1801) or standard DMEM/F12 media (Fisher; 10770245) supplemented with 1 % astrocyte growth supplement (ScienCell; 1852), 10 % fetal bovine serum (FBS, Sigma; F7524) and 1 % penicillin/streptomycin (pen/strep, Sigma; P4333) in T75 culture flasks (Corning) unless otherwise indicated in the figure legends. Given human astrocyte speciality media were approximately twice the cost of the standard cell culture media with supplements, we assessed the levels of sCX3CL1 using both media, with the only difference being the base media. Variations in the levels of sCX3CL1 released from the astrocytes between experiments can be primarily attributed to this reason and are highlighted accordingly in the figure legends. The cells were grown for 14 days until 90 % confluent and then re-plated in six- or 24-well plates and used when 80 % confluent.

### Quantitative polymerase chain reaction (Q-PCR)

For mRNA expression of CX3CL1, human astrocytes were grown in six-well plates until 80 % confluent. Prior to stimulation, cells were starved in serum-free media for 3 h. The cells were then pre-treated with either VX-702 (p38 inhibitor) or an IKKβ inhibitor in serum-free media for 30 min, after which IL-1β, TNF-α or IFN-γ was added and incubated for 3 or 18 h. After treatment, the media were removed and cells were washed twice with phosphate-buffered saline (PBS) in preparation for Q-PCR analysis. RNA extraction was performed using either Qiagen or Macherey-Nagel methods as described by the manufacturers. Using Qiagen kits, the cell pellets were suspended in RLT lysis buffer (Qiagen Hilden, Germany) and then frozen at −80 °C. RNA was isolated using RNeasy mini kit (Qiagen, 74104). To eliminate genomic DNA, an on-column DNase digestion was carried out with the RNase-free DNase set (Qiagen 79254). After reverse transcription of mRNA (10 min at 25 °C; 120 min at 37 °C; 5 s at 85 °C) using the High-Capacity cDNA Reverse Transcription Kit (Applied Biosystems, 4368814), RT-PCR was performed with the 7900HT Fast Real-Time PCR System (Applied Biosystems, 4329001) according to the Standard Thermal Cycler Protocol (2 min at 50 °C; 10 min at 95 °C; 40 cycles of 15 s at 95 °C and 1 min at 60 °C). The threshold was set manually for all samples. The analysis was performed with the SDS 2.3 software. For the Macherey-Nagel method (Macherey-Nagel; 740955), cells were suspended in RA1 buffer with 1 % beta-mercaptoethanol and frozen at −80 °C. To eliminate genomic DNA, an on-column DNase digestion was carried out with the RNase-free DNase. After reverse transcription of mRNA (10 min at 25 °C; 120 min at 37 °C; 5 s at 85 °C) using the High-Capacity cDNA Reverse Transcription Kit (Applied Biosystems, 4368814), RT-PCR was performed according to the Standard Thermal Cycler Protocol (2 min at 50 °C; 10 min at 95 °C; 40 cycles of 15 s at 95 °C and 1 min at 60 °C). TaqMan Gene Expression Assays using TaqMan probes of human CX3CL1 (Applied Biosystems; Hs00171086_M1) and HPRT (Applied Biosystems; Hs01003267_M1) (all FAM dye labelled) were used. The relative expression of CX3CL1 to the reference gene HPRT was determined. Each condition was run in triplicates, and the experiments were repeated independently three times unless otherwise indicated in the figure legends.

### Enzyme-linked immunosorbent assay (ELISA)

All cells were starved in serum-free media before treatments. Human astrocytes were pre-treated with inhibitors for 30 min followed by 18-h treatment with cytokines as indicated in the figure legends. Media were then collected, and cells were lysed in PTxE lysis buffer (1 mM EDTA and 1 % Triton-X in PBS) and frozen at −20 °C. Soluble fractalkine (sCX3CL1) levels in the supernatant and total cellular content were measured with human CX3CL1 ELISA kit according to the manufacturer’s instructions (R&D Systems; DY365). This DuoSet detects the N-terminal chemokine domain of human fractalkine. The level of detection for fractalkine indicated by the manufacturers is 0.63–20 ng/ml, although we noted significant and detectable changes in colour at lower levels. Briefly, 96-well ELISA plates (Thermo Scientific; 95029780) were coated overnight at room temperature with capture antibodies diluted in PBS. The plates were washed three times with wash buffer (0.05 % Tween 20 (Sigma; P7949), PBS, pH 7.4) and then blocked for 1 h at room temperature with the appropriate reagent diluent. The plates were then washed three times with wash buffer, and any remaining buffer was removed from the wells by aspiration. A standard curve was prepared using serial dilutions of the recombinant protein diluted in the appropriate reagent diluents. The samples and standards were then incubated in the antibody-coated ELISA plate for 2 h at room temperature. The plate was then washed three times with wash buffer, and detection antibody (diluted in reagent diluent) was added to each well for 2 h. Following three more washes, streptavidin-HRP diluted in reagent diluents was added to each well and incubated for 20 min at room temperature, protected from light. After an additional three washes, the wells were incubated with substrate solution (R&D systems; DY999) for 15 min at room temperature protected from light. The colour reaction was stopped with the addition of 1 M H_2_SO_4_, and absorbance was read immediately using a plate reader at 450 nm (Labsystem Multiskan). The standard curve was calculated by plotting the standards against the absorbance values, and the cytokine levels were measured in pg/ml.

### Immunocytochemistry

Human astrocytes were plated on glass coverslips in six-well plates (VWR) and cultured for at least 24 h until 80 % confluent. The cells were then washed with PBS and fixed with 4 % paraformaldehyde (PFA) for 10 min at room temperature. Permeabilization and nonspecific binding was reduced by incubating cells for 1 h at room temperature in blocking buffer (PBS supplemented with 1 % bovine serum albumin (BSA) and 0.1 % Triton-X 100). For all antibody incubation and wash steps, diluted blocking buffer (PBS supplemented with 0.5 % BSA and 0.01 % Triton-X 100) was used. The cells were incubated overnight at 4 °C with the rabbit anti-CX3CL1, which binds the N-terminal chemokine domain (1:500, eBioscience; 14-7986); washed three times; and incubated with anti-rabbit secondary antibody conjugated to Alex488 (1:1000; Invitrogen; A11008) for 1 h. After washing three times in PBS supplemented with 0.01 % Triton-X 100, the cells were then incubated with Hoechst nuclear stain (diluted 1:10,000 in PBS)(Invitrogen; H21486) for 10 min and washed twice again. The coverslips were then mounted on glass slides in Vectashield (Vector Labs) and the edges sealed with varnish. Samples were stored at 4 °C in the dark until imaged. The cells were visualized with a confocal microscope (Leica SP8).

### Statistical analysis

All statistical analysis was performed using GraphPad Prism 5. In experiments where three or more groups were compared, an ordinary one-way analysis of variance (ANOVA) was performed and was followed by post hoc tests. Tukey’s post hoc test was used for experiments where all columns were compared to each other. Student’s *t* test was used to compare the means between two groups. Detailed data analysis methods are provided in the “Methods” section, figure legends and “Results” section.

## Results

### CX3CL1 release from human astrocytes is stimulated by pro-inflammatory cytokines

**Fig. 1:**
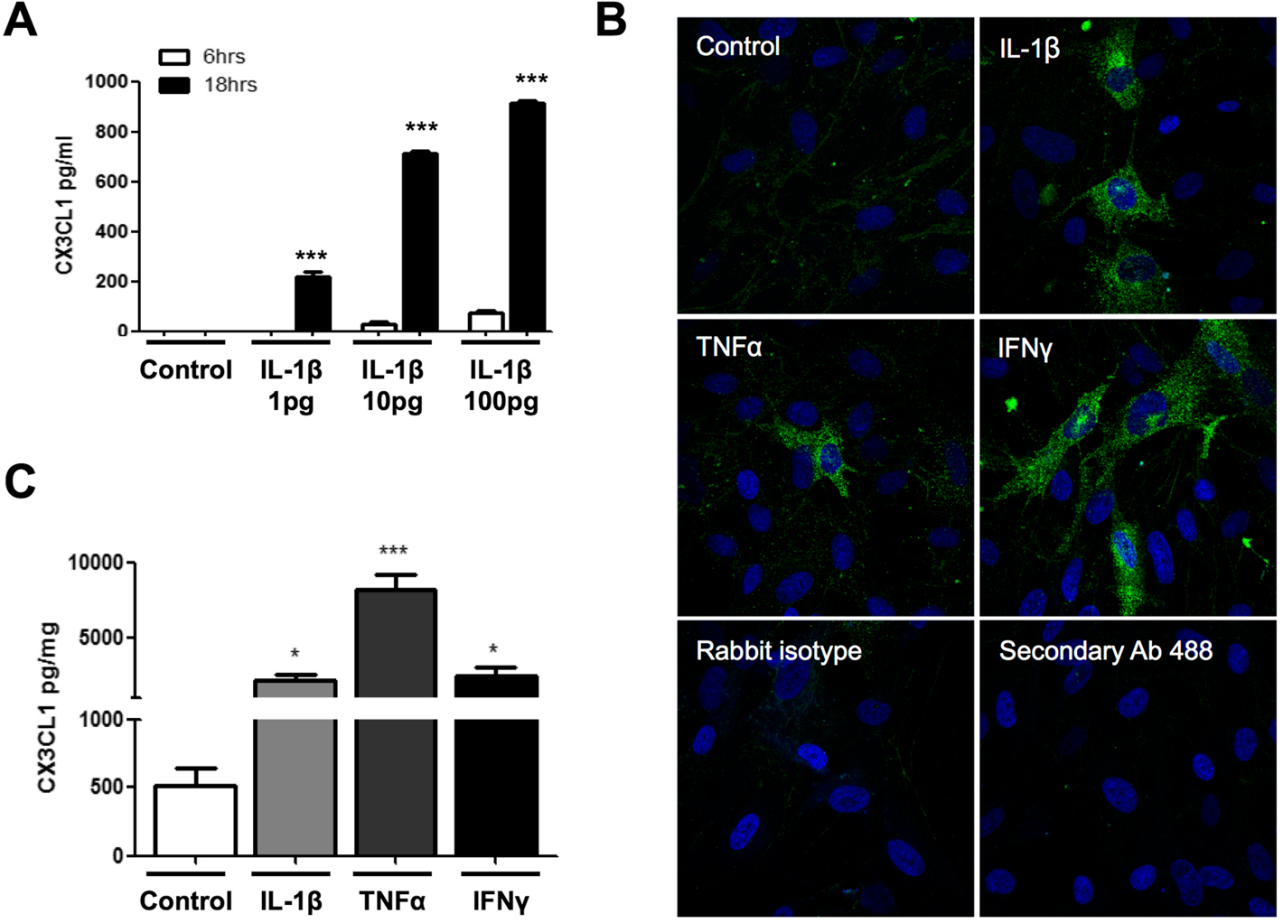
Soluble fractalkine (sCX3CL1) release from human astrocytes. **a** Human astrocytes (grown in speciality media) were treated with IL-1β (1 pg/ml, 10 pg/ml and 100 pg/ml) for 6 or 18 h, and the levels of sCX3CL1 in the media were quantified by ELISA. The levels of CX3CL1 were significantly higher after 18-h treatments compared to 6 h. All values expressed as averages ± SEM; *n* = 4, each condition done in duplicate. One-way ANOVA and Tukey’s post hoc test; ***p < 0.001 compared to control. **b** Immunostaining of up-regulated CX3CL1 on human astrocytes (grown in speciality media) when treated with IL-1β (100 pg/ml), TNF-α (10 ng/ml) and IFN-γ (10 ng/ml) for 18 h. **c** Quantification of total cellular content of CX3CL1 following cytokine stimulation. Human astrocytes (grown in standard media) were treated with a IL-1β (100 pg/ml), TNF-α (10 ng/ml) and IFN-γ (10 ng/ml) for 18 h. Following stimulation, cells were lysed with PTxE lysis buffer and the levels of sCX3CL1 were quantified by ELISA. Data presented as mean ± SEM, *n* = 4, unpaired *t* test, *p < 0.05, ***p < 0.001 vs. corresponding control. In all cases, cells were serum starved 3 h before treatments

As a result of the low expression of CX3CL1 on astrocytes, its functional expression has not been well characterized to date. In agreement with previous observations [3, 6], astrocytes showed little to no release of sCX3CL1 into the media after 6 and 36 h of serum starvation, suggesting minimal surface expression and subsequent cleavage of CX3CL1 under basal conditions (data not shown). In contrast, the treatment of human astrocytes with IL-1β induced a concentration- and time-dependent increase in the levels of sCX3CL1 (Fig. 1a). Specifically, human astrocytes treated with IL-1β for 6 h showed no significant increase in the levels of sCX3CL1, whereas 18-h treatment with IL-1β at 1 pg/ml (220.5 ± 17.5), 10 pg/ml (711.8 ± 12.8) and 100 pg/ml (911.5 ± 10.0) significantly increased the levels of sCX3CL1 (Fig. 1a). Immunocytochemistry also showed the treatment of human astrocytes with IL-1β (100 pg/ml), TNF-α (10 ng/ml) or IFN-γ (10 ng/ml) increased the levels of CX3CL1 in human astrocytes, using an antibody directed against the chemokine N-terminal domain of CX3CL1 (eBioscience; 14-7986) (Fig. 1b). In these experiments, under control conditions, we noted little to no cellular expression of fractalkine, in agreement with the ELISA data. Treatment with the cytokines, IL-1β, TNF-α and IFN-γ, increased the levels of fractalkine that appeared within the cell, with no evident appearance at the cell membrane. We propose that rapid shedding of fractalkine once it reaches the membrane may result in a low/no detection of fractalkine at the cell membrane. In support, we also examined the levels of fractalkine in the cell and found that treatment with the cytokines, IL-1β, TNF-α and IFN-γ, increased significantly the levels of fractalkine in the cell lysates (Fig. 1c). This data is in line with other studies suggesting low levels of fractalkine in astrocytes under basal conditions, which increases with inflammatory stimuli [6, 40].

### IL-1β, TNF-α and IFN-γ increase the levels of CX3CL1 mRNA

**Fig. 2:**
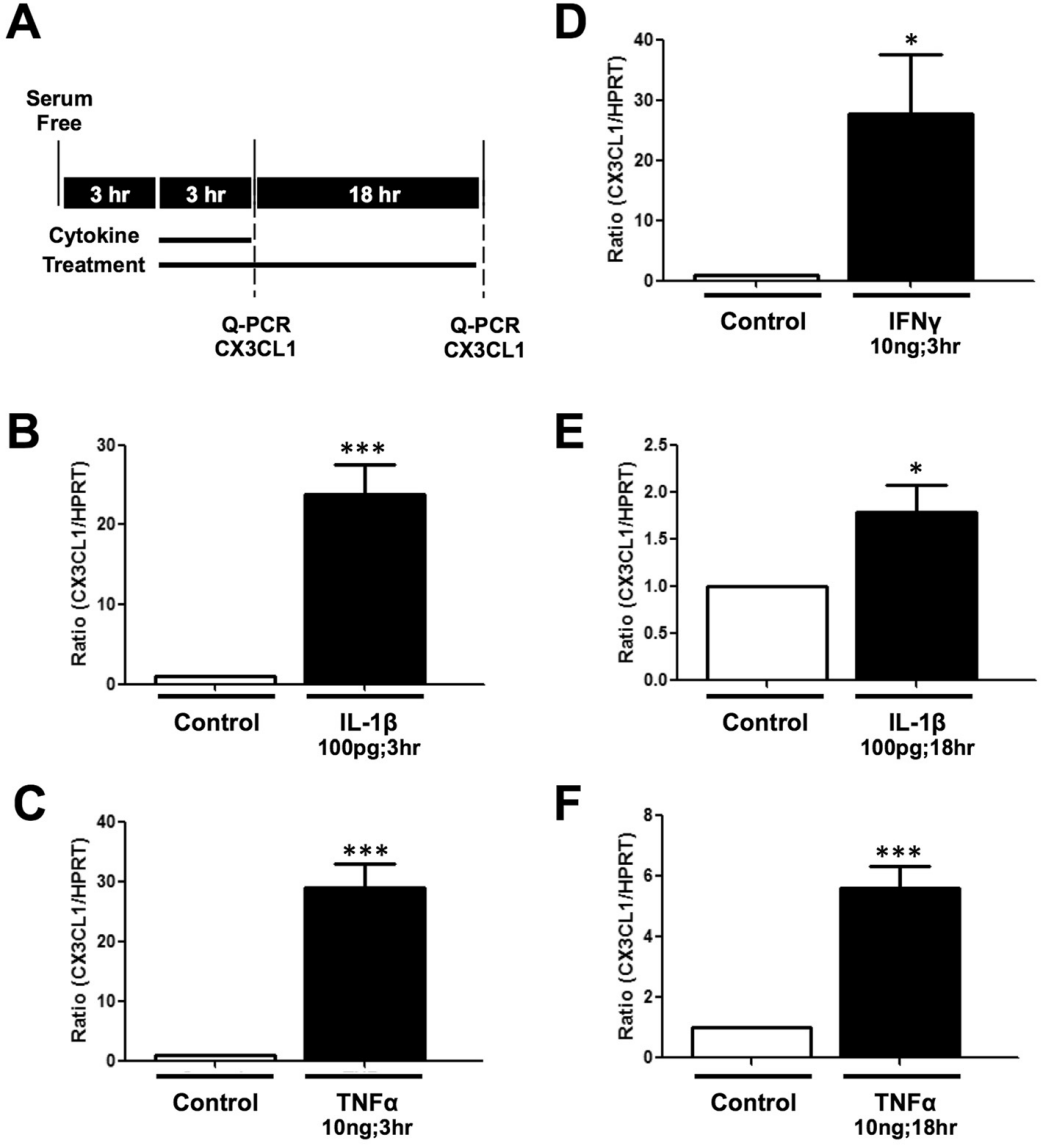
IL-1β, TNF-α and IFN-γ induce CX3CL1 mRNA synthesis in human astrocytes. **a ** Experimental timeline and treatments are shown. Human astrocytes were serum starved for 3 h prior to stimulation with **b**, **e** IL-1β (100 pg/ml), **c**, **f** TNF-α (10 ng/ml) or **d** IFN-γ (10 ng/ml) for either 3 h (grown in standard media) or 18 h (grown in speciality media). All graphs show significant increase in CX3CL1 mRNA levels after 3 and 18 h. Data presented as mean ± SEM, *n* = 4–8, unpaired *t* test, *p < 0.05, ***p < 0.001 vs. corresponding control

To determine whether IL-1β, TNF-α and IFN-γ alter the levels of CX3CL1 by increasing protein synthesis, we examined their effect on CX3CL1 mRNA using Q-PCR (Fig. 2a). Treatment of human astrocytes with IL-1β (100 pg/ml, Fig. 2b), TNF-α (10 ng/ml, Fig. 2c) and IFN-γ (10 ng/ml, Fig. 2d), for 3 h, significantly increased the levels of CX3CL1 mRNA in all three cases, by 20-fold or more. These effects appeared transient in so far as cytokine treatment for 18 h increased the levels of CX3CL1 mRNA but not to the same extent as 3 h. In particular, IL-1β treatment significantly increased the levels of CX3CL1 mRNA but only by approximately twofold (Fig. 2e), and treatment for 18 h with TNF-α increased the levels of CX3CL1 mRNA by approximately fivefold compared to control (Fig. 2f). As an additional note, we confirmed these cytokines did not cause cell death using MTT assays and noted IL-1β and TNF-α caused a modest increase in cell viability (Additional file 1: Figure S1B). Overall, these results show that the cytokines IL-1β, TNF-α and IFN-γ increase the expression of CX3CL1 by likely promoting mRNA and protein synthesis, without overtly affecting cell survival.

### Matrix metalloprotease inhibitors attenuate IL-1β-, TNF-α- and IFN-γ-induced levels of CX3CL1 in human astrocytes

**Fig. 3:**
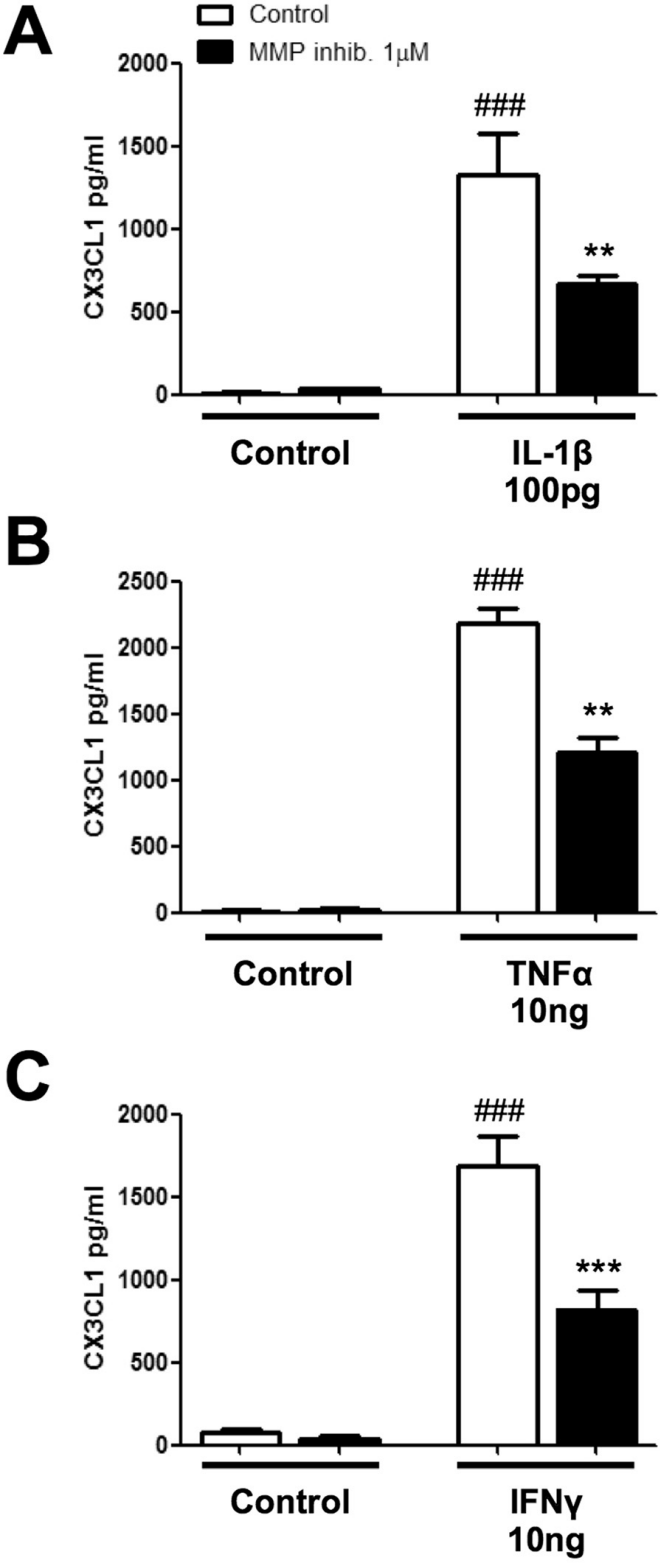
Inhibition of matrix metalloprotease’s attenuates sCX3CL1 release from human astrocytes. Human astrocytes (grown in speciality media) were pre-treated with a pan MMP inhibitor (MMP inhib.) Marimastat (1 μM) for 30 min. Cells were then stimulated with either **a** IL-1β (100 pg/ml), **b** TNF-α (10 ng/ml) or **c** IFN-γ (10 ng/ml) for 18 h. All groups were serum starved 3 h before treatments. Quantification of CX3CL1 ELISA revealed a significant decrease in CX3CL1 when cells were pre-treated with Marimastat for all treatment groups. ###*p* < 0.001 compared to own control; ***p < 0.001 and **p < 0.05 compared to the matched treated group. All values expressed as averages ± SEM; *n* = 3–5, each condition done in duplicate (one-way ANOVA and Tukey’s post hoc test)

Matrix metalloproteases (MMPs) are a family of proteases that are capable collectively of degrading proteins in the extracellular matrix (ECM). MMPs are also involved in the cleavage of cell surface receptors and ligands such as cytokines and chemokines [49]. Thus, MMPs can play important roles in regulating cellular processes such as cell death and inflammation [49]. Notably, however, the ability of MMPs to cleave the CX3CL1 ligand is less well studied in glial cells, particularly in astrocytes. To determine whether MMPs play a role in the release of the CX3CL1 ligand from human astrocytes, the soluble levels of CX3CL1 were measured in astrocyte-conditioned media using ELISA. Human astrocytes were pre-treated with a pan MMP inhibitor, RS-130830 (1 μM for 30 min), prior to 18-h treatment with the pro-inflammatory stimuli; IL-1β (100 pg/ml), TNF-α (10 ng/ml) or IFN-γ (10 ng/ml). Treatment with IL-1β (1328.0 ± 253.8) (Fig. 3a), TNF-α (2185.0 ± 113.3) (Fig. 3b) and IFN-γ (1687 ± 181.2) (Fig. 3c) increased the levels of sCX3CL1 after 18 h, compared to control (###*p* < 0.001 *n* = 3; one-way ANOVA and Tukey’s post hoc test). Importantly, pre-treatment with a pan MMP inhibitor, RS-130830, attenuated the CX3CL1-induced increase by IL-1β (666.8 ± 46.8; **p < 0.01), TNF-α (1207.0 ± 108.4; ***p < 0.001) and IFN-γ (821.0 ± 110.9; *p < 0.05), respectively, compared to the matched treated group (one-way ANOVA and Tukey’s post hoc test) (Fig. 3a–c). Notably, the MMP inhibitor, RS-130830, had no effect on cell viability (Additional file 1: Figure S1C). These results suggest that human astrocytes shed the CX3CL1 chemokine in response to IL-1β, TNF-α and IFN-γ and that pre-treatment with a pan MMP inhibitor significantly reduces this response.

### ADAM10 inhibitor reduces pro-inflammatory cytokine-mediated increase of CX3CL1 in human astrocytes

**Fig. 4:**
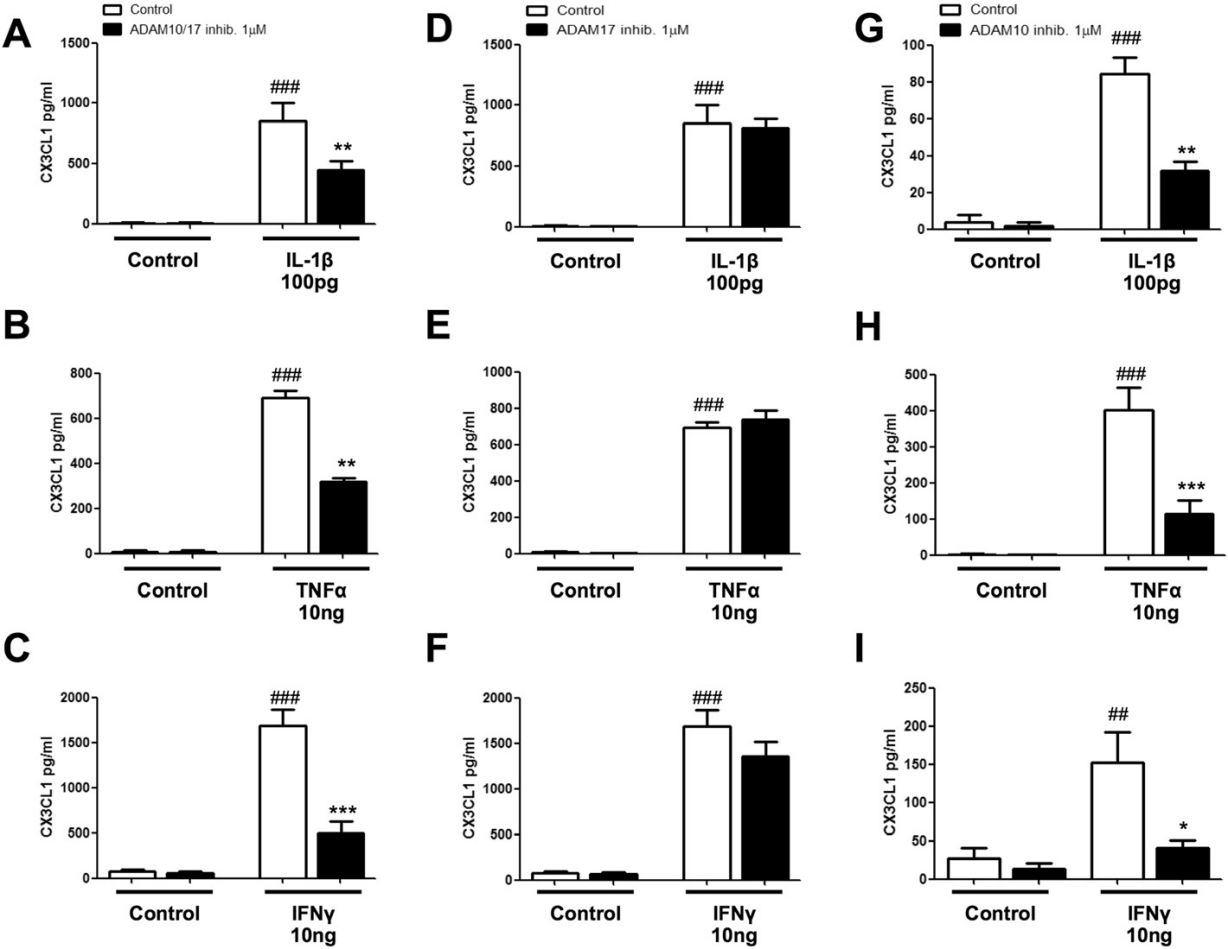
ADAM10 inhibitor attenuates IL-1β-, TNF-α- and IFN-γ-induced sCX3CL1 release. Human astrocytes (**a**–**f** grown in speciality media, **g**–**i** grown in speciality media) were serum starved for 3 h and pre-treated with a combination of the ADAM10/17 inhibitor (BMS-561392; 1 μM), specific TACE inhibitor (BMS-566394; 1 μM) or specific ADAM10 inhibitor (GI 254023X; 1 μM) for 30 min. Cells were then stimulated with either IL-1β (100 pg/ml) (**a**, **d**, **g**), TNF-α (10 ng/ml) (**b**, **e**, **h**) or IFN-γ (10 ng/ml) (**c**, **f**, **i**) for 18 h. Quantification of CX3CL1 ELISA revealed a significant decrease in sCX3CL1 when pre-treated with the ADAM10/17 inhibitor and specific ADAM10 inhibitor. No significant difference was seen with the specific ADAM17 inhibitor. ###*p* < 0.001 compared to own control; *p < 0.05, **p < 0.01 and ***p < 0.001 compared to the matched treated group. Values expressed as averages ± SEM; *n* = 4–8, each condition done in duplicate (one-way ANOVA and Tukey’s post hoc test)

ADAM10 and ADAM17 proteases have also been implicated in the cleavage of CX3CL1 from the cell surface of ECV-304 cells and fibroblasts, with ADAM10 involved in constitutive shedding of the ligand [23, 30]. To our knowledge, it is unknown whether the same proteinases are used to cleave the CX3CL1 on astrocytes. We again noted low levels of CXC3L1 in conditioned media taken from human astrocytes, suggesting low levels of expression in these cells under control conditions and which also suggests the amount of constitutive shedding from human astrocytes is low. Next, to investigate whether ADAM10 and/or ADAM17 proteases are involved in the inducible shedding of CX3CL1 from human astrocytes under stimulated conditions, these cells were pre-treated for 30 min with the dual ADAM10/ADAM17 (BMS-561392) inhibitor, the selective ADAM17 inhibitor (BMS-566394) or the selective ADAM10 inhibitor (TOCRIS; GI 254023X) before treatment with the pro-inflammatory recombinant cytokines, IL-1β (100 pg/ml), TNF-α (10 ng/ml) or IFN-γ (10 ng/ml). As expected, IL-1β (84.6 ± 8.7, Fig. 4a), TNF-α (400.6 ± 63.06, Fig. 4b) and IFN-γ (163.5 ± 49.05, Fig. 4c) significantly increased the levels of CX3CL1 (###*p* < 0.001 compared to control). Importantly, pre-treatment with the ADAM10/ADAM17 dual inhibitor significantly decreased the levels of CX3CL1 induced by IL-1β (663.7 ± 39.5, Fig. 4a), TNF-α (600.3 ± 30.2, Fig. 4b) and IFN-γ (847.8 ± 146.6, Fig. 4c) (***p < 0.001, **p < 0.01 compared to the matched treated group). In contrast, the selective inhibitor for ADAM17 did not alter the levels of CX3CL1 induced by IL-1β (973.5 ± 64.3, Fig. 4d), TNF-α (944.2 ± 39.0, Fig. 4e) and IFN-γ (1776.0 ± 129.3, Fig. 4f). However, the specific ADAM10 inhibitor also attenuated the IL-1β (84.5 ± 8.7 vs. 32.0 ± 5.0, Fig. 4g), TNF-α (401.0 ± 63.1 vs. 115 ± 36.0, Fig. 4h) and IFN-γ (163.5 ± 49.1 vs. 47.9 ± 8.6, Fig. 4i) induced levels of sCX3CL1 (**p < 0.01, *p < 0.05 compared to the matched treated group). During our experiments, we noted variations in the absolute levels of CX3CL1, between experiments. These can be linked to the different batches of human astrocytes used, the age/passage number of these cells and the use of speciality or standard media. Importantly, however, while the absolute levels of CX3CL1 differed between experiments, the effects of the cytokines IL-1β, TNF-α and IFN-γ were similar as the effects of the inhibitors used. To convince ourselves further of a role for ADAM10 in the inducible increase in the levels of CX3CL1, we replicated experiments examining the effects of the ADAM10 inhibitor by co-stimulating astrocytes with TNF-α and IFN-γ (1 ng each). In this experiment, we still found that the ADAM10 inhibitor significantly attenuated TNF-α/IFN-γ-induced levels of fractalkine (19,948 ± 2357 vs. 5208 ± 1569) (Additional file 1: Figure S1A) (***p < 0.001, compared to the matched treated group). The effects of this ADAM10 inhibitor were also not associated with any significant changes in cell viability (Additional file 1: Figure S1C). Of interest, we also examined the effect of IL-1β, TNF-α and IFN-γ on the expression levels of ADAM10 in human astrocytes and found they did not alter the levels of either the pro- or active form of ADAM10 (Additional file 1: Figure S1D). Taken together, these data suggest ADAM10 plays a central role in the shedding of CX3CL1 from astrocytes and that the cytokines IL-1β, TNF-α and IFN-γ likely alter the enzymatic activity of ADAM10, rather than altering its level of expression, per se.

### Inhibition of the p38 MAP kinase attenuates IL-1β-, TNF-α- and IFN-γ-induced levels of sCX3CL1

**Fig. 5:**
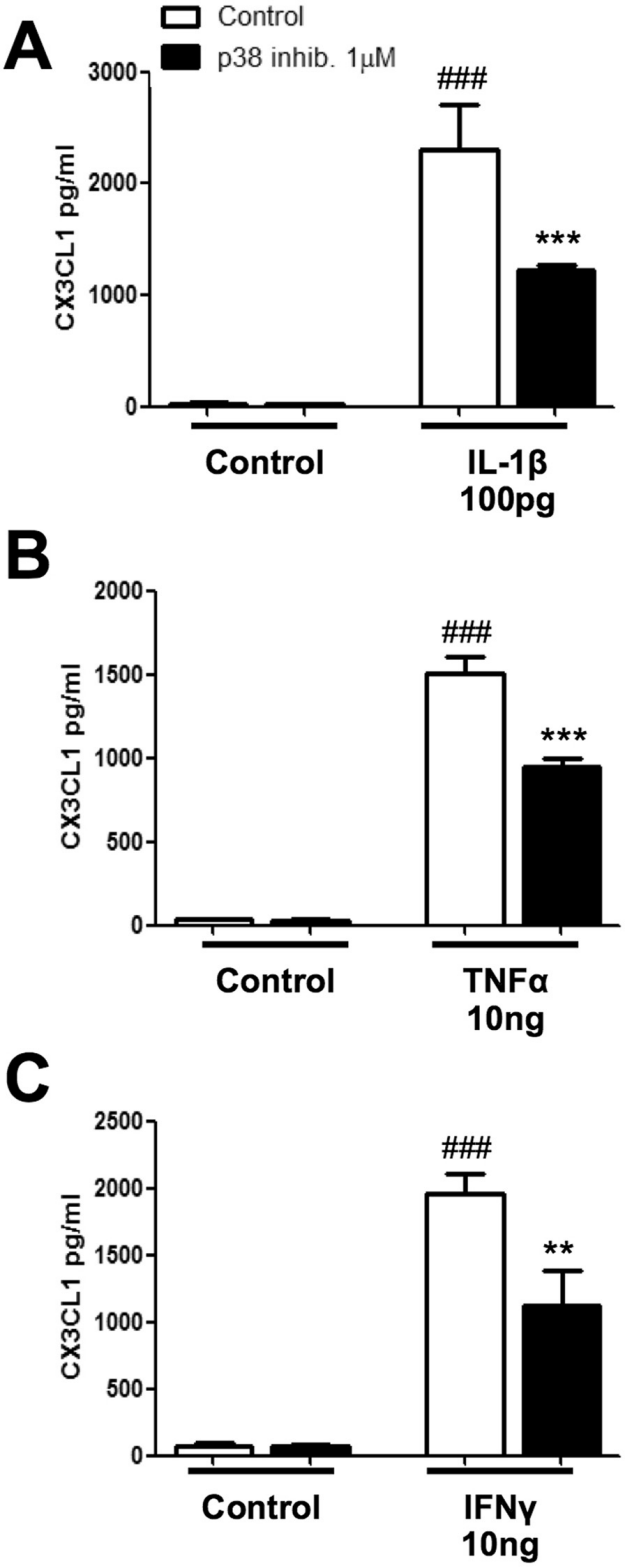
The p38 inhibitor (VX-702) reduces IL-1β-, TNF-α- and IFN-γ-induced sCX3CL1 release. Human astrocytes (grown in speciality media) were serum starved for 3 h and pre-treated with the p38 inhibitor, VX-702 (1 μM), for 30 min. Cells were then stimulated with either **a** IL-1β (100 pg/ml), **b** TNF-α (10 ng/ml) or **c** IFN-γ (10 ng/ml) for 18 h. Quantification of CX3CL1 ELISA revealed a significant decrease in sCX3CL1 when pre-treated with VX-702 for all treatment groups. ###*p* < 0.001 compared to own control; ***p < 0.001 and **p < 0.01 compared to the matched treated group. Values expressed as averages ± SEM; *n* = 3, each condition done in duplicate (one-way ANOVA and Tukey’s post hoc test)

Previous studies have demonstrated that the cytokines IL-1β, TNF-α and IFN-γ alter the levels of p38 in astrocytes [37, 44, 50–53]. The p38 inhibitor, VX-702, has been extensively used in the literature. VX-702 has been validated as a p38 inhibitor and has been clinically evaluated for the treatment of chronic inflammatory disorders (ClinicalTrials.gov Identifier NCT00395577) [54, 55]. Several studies have shown that p38 MAP kinase (MAPK) is required for phosphorylation and activation of ADAM10 and ADAM17 in peripheral cells [27, 30]. Therefore, to investigate if IL-1β, TNF-α and IFN-γ promote cleavage of CX3CL1 via a p38 MAPK-dependent pathway, we tested if the p38 MAPK inhibitor, VX-702 (TOCRIS; 3916), attenuated the increase of CX3CL1 levels induced by these pro-inflammatory cytokines. Human astrocytes were serum starved for 3 h prior to pre-treatment with the p38 inhibitor, VX-702 (1 μM; 30 min), and then incubated with IL-1β (100 pg/ml), TNF-α (10 ng/ml) or IFN-γ (10 ng/ml) for 18 h. In line with previous data [29, 56], the p38 MAPK inhibitor attenuated the IL-1β (2552 ± 91.18 vs. 1254 ± 36.46) (Fig. 5a), TNF-α (1475 ± 91.7 vs. 969.7 ± 49.07) (Fig. 5b) and IFN-γ (2033 ± 187.6 vs. 1118 ± 231.6) (Fig. 5c) induced levels of sCX3CL1 (***p < 0.001, **p < 0.01, compared to the matched treated group), without causing any changes in cell viability (Additional file 1: Figure S1C). This data may be likely explained by a required phosphorylation, preceding an ADAM10-mediated shedding of CX3CL1 from human astrocytes.

### IKKβ inhibitor attenuates pro-inflammatory cytokine-induced protein levels of CX3CL1 in human astrocytes

**Fig. 6:**
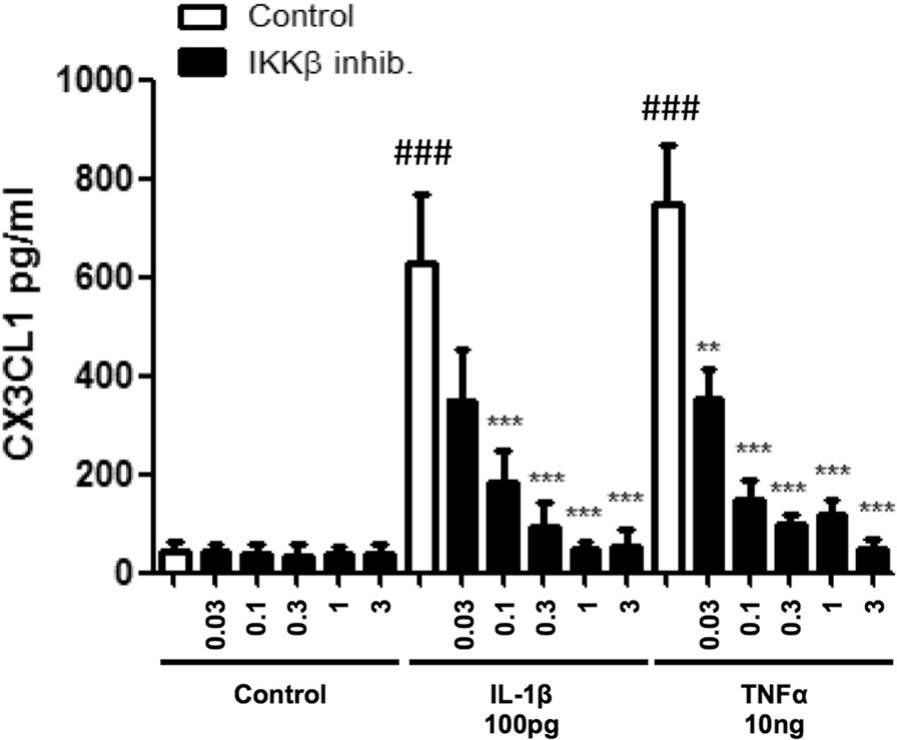
IKKβ inhibitor attenuates sCX3CL1 release. Human astrocytes (grown in speciality media) were serum starved for 3 h and pre-treated with the NF-kB inhibitor, TPCA-1 (IKKβ inhib.; 3, 1, 0.3, 0.1 and 0.03 μM), for 30 min. Cells were then stimulated with either IL-1β (100 pg/ml) or TNF-α (10 ng/ml) for 18 h. Quantification of CX3CL1 ELISA revealed a concentration-dependent decrease in sCX3CL1 release for both IL-1β- and TNF-α-treated groups. ###*p* < 0.001 compared to own control; **p < 0.01 and ***p < 0.001 compared to the matched treated group. Values expressed as averages ± SEM; *n* = 3 each condition done in duplicate. (one-way ANOVA and Tukey’s post hoc test)

The transcription factor NF-kB regulates the production of several pro-inflammatory cytokines [57]. Reciprocally, the cytokines IL-1β, TNF-α and IFN-γ alter NF-kB signalling [37, 42, 43, 53]. Thus, in order to further investigate the mechanism by which pro-inflammatory cytokines increase CX3CL1 levels in human astrocytes, effects of the NF-kB pathway inhibitor IKKβ (TPCA-1) were examined on the protein levels of CX3CL1 using ELISA. Human astrocytes were serum starved for 3 h before 30-min pre-treatment with the IKKβ (TPCA-1, 3, 1, 0.3, 0.1 and 0.03 μM) inhibitor, prior to the addition of IL-1β (100 pg/ml) or TNF-α (10 ng/ml) for 18 h. As demonstrated previously, IL-1β and TNF-α increased the levels of CX3CL1, which were significantly attenuated by the IKKβ inhibitor (TPCA-1, using 1 μM, 628.3 ± 138.5 vs. 47.7 ± 14.9 for IL-1β; 748.0 ± 120.0 vs. 116.4 ± 31.7 for TNF-α) (Fig. 6) (###*p* < 0.001 compared to own control; **p < 0.01 and ***p < 0.001 compared to the matched treated group). From this data, we conclude that the pro-inflammatory cytokines IL-1β and TNF-α increase the levels of sCX3CL1 by promoting NF-kB-dependent synthesis which results in subsequent enhanced shedding of CX3CL1 via an ADAM10-dependent mechanism.

## Discussion

### Summary of findings

In the current study, we investigated fractalkine (CX3CL1) expression and regulation in human astrocytes. We showed firstly that CX3CL1 is expressed in human astrocytes and is constitutively shed at low levels. On stimulation with pro-inflammatory cytokines such as IL-1β, TNF-α and IFN-γ, this low level of sCX3CL1 is significantly up-regulated. We noted that a broad-spectrum MMP inhibitor attenuated this increase in CX3CL1. Using a dual inhibitor of ADAM10/ADAM17 and selective ADAM10 and ADAM17 inhibitors, we showed further that ADAM10 is the main protease responsible for the cytokine-mediated inducible shedding of sCX3CL1. We also found these cytokines stimulated the mRNA levels of CX3CL1 suggesting a mechanism involving increased mRNA and protein synthesis. Using a p38 MAPK inhibitor, we demonstrated that the pro-inflammatory cytokine-mediated increase in sCX3CL1 likely involved a phosphorylation event mediated by p38 MAPK. An inhibitor of IKKβ also attenuated the increase in sCX3CL1 induced by the pro-inflammatory cytokines IL-1β and TNF-α, suggesting involvement of the NF-kB pathway. Taken together, the data indicates that pro-inflammatory cytokines regulate CX3CL1 expression on human astrocytes and that ADAM10 is the main protease involved in the liberation of sCX3CL1 from human astrocytes.

### ADAM10 regulates cytokine-mediated levels of sCX3CL1 in astrocytes

Both ADAM10 and ADAM17 have been implicated in the development of neurodegenerative diseases such as Alzheimer’s disease [25, 58, 59]. Studies have also shown that ADAM10 expression is widespread in the CNS compared to a more restricted ADAM17 expression [31]. A more ubiquitous expression of ADAM10 would suggest this enzyme may be responsible for the constitutive basal processing of ligands such as CX3CL1 [31, 59]. In contrast, a low expression of ADAM17 appears to coincide with its proposed involvement in inducible proteolytic processing. For example, previous studies show that pro-inflammatory cytokines, such as CXCL12, can regulate cleavage of the CX3CL1 ligand from neurons [12]. The treatment of neurons with CXCL12 stimulates the expression of ADAM17 causing an increase in sCX3CL1 [12]. Studies also show that ADAM10 is responsible for constitutive neuronal CX3CL1 cleavage [23]. In contrast, our studies on human astrocytes would suggest that ADAM10 is responsible for the cytokine-mediated proteolytic processing of sCX3CL1. In support of our findings, siRNA knockdown of ADAM-10 (but not ADAM17), in a human adult brain endothelial cell line, was shown to significantly reduce sCX3CL1, following pro-inflammatory cytokine treatment [60]. This finding raises the possibility that differential regulation of ADAM10 and ADAM17 may be cell type dependent and provide a mechanism for regulating different cellular responses to the same stimuli.

### Involvement of p38 MAPK in the regulation of ADAM10

Similar to studies investigating the release of CX3CL1 from neurons, we show here that the shedding of CX3CL1 occurs both constitutively and in response to inflammatory stimuli in astrocytes [23, 30]. We find, in particular, that ADAM10 likely regulates pro-inflammatory cytokine-induced levels of CX3CL1 in astrocytes. Notably, several other ligands are shed by ADAM10, such that cleavage by this protease is likely controlled by multiple regulatory mechanisms [61]. The cytosolic tail of ADAM10, as well as other ADAM proteases, contains many interaction motifs for signalling and adapter proteins, which could conceivably regulate protease activity [61]. Indeed, studies have shown that downstream signalling molecules such as p38 interact with the cytoplasmic domain of ADAM10 and regulate ADAM-dependent ligand shedding [28, 30, 62]. Popular thought is that constitutive shedding is governed by p38 [61, 63]. In neurons, it appears that p38 regulates the constitutive shedding of CX3CL1 via ADAM10 [27, 28]. Here, we show that, in astrocytes, inhibition of p38 caused a significant decrease in the levels of sCX3CL1 induced by treatment with pro-inflammatory cytokines. It is therefore reasonable to suggest that, in astrocytes, p38 via ADAM10 may enhance cleavage of CX3CL1 and/or that a p38 signalling pathway is required for increased production of this ligand.

### Functions of astrocyte-derived CX3CL1

CX3CL1 signalling is crucial for proper development during brain maturation [64], synaptic plasticity [65, 66], neuroprotection [67, 68] and neurotoxicity [69, 70]. More specifically, sCX3CL1 has the capability of enhancing microglial response to CNS damage, thereby promoting a pro-inflammatory environment [69]. However, membrane-tethered CX3CL1 can also serve as an adhesion molecule, thereby anchoring cells to the extracellular matrix and preventing them from migrating [5]. In a myelin oligodendrocyte glycoprotein (MOG)-induced experimental autoimmune encephalomyelitis (EAE) rat model of MS, neuronal CX3CL1 was reported unchanged and remained at control levels [16]. However, at the sites of inflammation and surrounding active lesions, there is an increase in astrocyte-associated CX3CL1 along with increased expression of CX3CR1 on microglia [16]. A possible role for astrocyte-derived CX3CL1 may be to enhance CX3CR1 signalling on microglia and at the same time promote levels of neuronal CX3CL1 as an attachment factor. This dual role of CX3CL1 within the CNS may thus serve to protect the surrounding tissue from damaged neurons while also mobilizing microglia and astrocytes to eliminate the damaged tissue.

### Studies using CX3CL1 as a drug target—lessons learned so far

CX3CL1 or CX3CR1 gene-disrupted animals are able to mature to adulthood. However, CX3CR1^−/−^ mice have more synapses, a reduced number of microglia in the hippocampus and delayed maturation of functional glutamate receptors [64, 71]. Disruption to CX3CL1/CX3CR1 from conception also causes a differential response to fractalkine treatment in comparison to wild-type animals following inflammatory and neurodegenerative insults [67, 69]. In contrast, ADAM10 knockout animals are embryonic lethal [72]. The genetic manipulation of CX3CL1 signalling has to date led to contradictory findings in mouse models of peritonitis regarding its role in immune defence [73, 74]. In particular, studies have shown that monocyte extravasation is not affected in CX3CR1-deficient mice, suggesting that CX3CR1 signalling is not necessary for invading monocytes [73]. In contrast, CX3CR1-mediated signalling is demonstrated to be essential for macrophages in mounting defence against bacterial infection [74]. As the CX3CL1-CX3CR1 interaction appears exclusive, in contrast to other chemokines, this specificity has been considered as worthy of exploitation for drug development. Previous studies involving either CX3CL1- or CX3CR1-deficient mice have, however, led to contradictory findings, where CX3CL1 is suggested as neuroprotective or neurotoxic. Studies, for example, on middle cerebral artery occlusion (MCAO) in CX3CL1-deficient mice exhibited smaller infarct lesions and had lower mortality rates [75]. However, administration of exogenous CX3CL1, to CX3CL1-deficient mice, post ischemia, resulted in neurotoxicity and increased infarct size [75]. Notably, these neurotoxic effects of CX3CL1 were not observed in CX3CR1-deficient mice [75]. Studies, in contrast, also show that CX3CL1 administered to wild-type rats, just prior to MCAO, elicits long-lasting neuroprotective effects [76]. In addition to this, a narrow therapeutic concentration range for CX3CL1 appears to exist, where low concentrations are neuroprotective (15–70 pM) with higher levels (>150 pM) becoming toxic [76]. Therefore, from these studies, it seems that the concentration of CX3CL1 and its administration pre or post insult, as well as activity of the endogenous CX3CL1 signalling pathway, determine the efficacy of CX3CL1 in diseased models.

## Conclusions

Here, we find that CX3CL1 shedding from astrocytes is mediated by pro-inflammatory cytokines via an ADAM10-dependent pathway, where p38 MAPKs as well as NF-kB signalling may also play a role. This study suggests that the regulation of CX3CL1 in glial cells may be an important process during inflammatory events.

## Additional file

Additional file 1: Figure S1. (A) Soluble fractalkine levels are attenuated with ADAM10 inhibition following co-stimulation with TNF-α and IFN-γ. Astrocytes (grown in standard media) were pre-treated with the ADAM10 inhibitor (GI 254023X; 1 μM), for 30 min. Cells were then co-stimulated with TNF-α (1 ng/ml) and IFN-γ (1 ng/ml) for 18 h. Quantification of CX3CL1 ELISA revealed a significant decrease in soluble fractalkine when pre-treated with the ADAM10 inhibitor. ###*p* < 0.001 compared to own control and ****p* < 0.001 compared to the matched treated group (one-way ANOVA and Tukey’s post hoc test). Values expressed as averages ± SEM; *n* = 4, duplicates. (B, C) Changes in the levels of sCX3CL1 are not associated with cell death. Astrocytes were treated with (B) IL-1β (100 pg/ml), TNF-α (10 ng/ml) and IFN-γ (10 ng/ml) or (C) a pan MMP inhibitor (MMP inhib.) Marimastat (1 μM), specific ADAM10 inhibitor (GI 254023X; 1 μM) and the p38 inhibitor, VX-702 (1 μM), all for 18 h. Cell viability was analysed using MTT assay. A significant increase in cell viability is seen with IL-1β and TNF-α. ***p* < 0.01 and ****p* < 0.001; one-way ANOVA and Tukey’s post hoc test. Values expressed as averages ± SEM; *n* = 5, triplicates. (D) Cytokines do not increase protein levels of the active form of ADAM10. Astrocytes were treated with IL-1β (100 pg/ml), TNF-α (10 ng/ml) and IFN-γ (10 ng/ml) for 18 h before western blotting (representative of three independent experiments). In all cases, human astrocytes were serum starved for 3 h before treatments.

## Abbreviations

ADAM: A disintigrin and metalloprotease
CNS: Central nervous system
CX3CL1: Fractalkine
CX3CR1: Fractalkine receptor
DMSO: Dimethyl sulfoxide
EAE: Experimental autoimmune encephalomyelitis
IFN-γ: Interferon gamma
IL-1β: Interleukin-1 beta
MAPK: Mitogen-activated protein kinase
MMP: Matrix metalloprotease
MOG: Myelin oligodendrocyte glycoprotein
NF-kB: Nuclear factor kappa b
sCX3CL1: Soluble fractalkine
TNF-α: Tumour necrosis factor alpha
