# Supplementary material for: Fractalkine shedding is mediated by p38 and the ADAM10 protease under pro-inflammatory conditions in human astrocytes
Source: J Neuroinflammation. 2016 Aug 22;13(1):189. doi: 10.1186/s12974-016-0659-7 (PMC4994207; doi:10.1186/s12974-016-0659-7)
Supplement: Additional file 1: Figure S1. — (A) Soluble fractalkine levels are attenuated with ADAM10 inhibition following co-stimulation with TNF-α and IFN-γ. Astrocytes (grown in standard media) were pre-treated with the ADAM10 inhibitor (GI 254023X; 1 μM), for 30 min. Cells were then co-stimulated with TNF-α (1 ng/ml) and IFN-γ (1 ng/ml) for 18 h. Quantification of CX3CL1 ELISA revealed a significant decrease in soluble fractalkine when pre-treated with the ADAM10 inhibitor. ###p < 0.001 compared to own control and ***p < 0.001 compared to the matched treated group (one-way ANOVA and Tukey’s post hoc test). Values expressed as averages ± SEM; n = 4, duplicates. (B, C) Changes in the levels of sCX3CL1 are not associated with cell death. Astrocytes were treated with (B) IL-1β (100 pg/ml), TNF-α (10 ng/ml) and IFN-γ (10 ng/ml) or (C) a pan MMP inhibitor (MMP inhib.) Marimastat (1 μM), specific ADAM10 inhibitor (GI 254023X; 1 μM) and the p38 inhibitor, VX-702 (1 μM), all for 18 h. Cell viability was analysed using MTT assay. A significant increase in cell viability is seen with IL-1β and TNF-α. **p < 0.01 and ***p < 0.001; one-way ANOVA and Tukey’s post hoc test. Values expressed as averages ± SEM; n = 5, triplicates. (D) Cytokines do not increase protein levels of the active form of ADAM10. Astrocytes were treated with IL-1β (100 pg/ml), TNF-α (10 ng/ml) and IFN-γ (10 ng/ml) for 18 h before western blotting (representative of three independent experiments). In all cases, human astrocytes were serum starved for 3 h before treatments. [file 12974_2016_659_MOESM1_ESM.pdf]

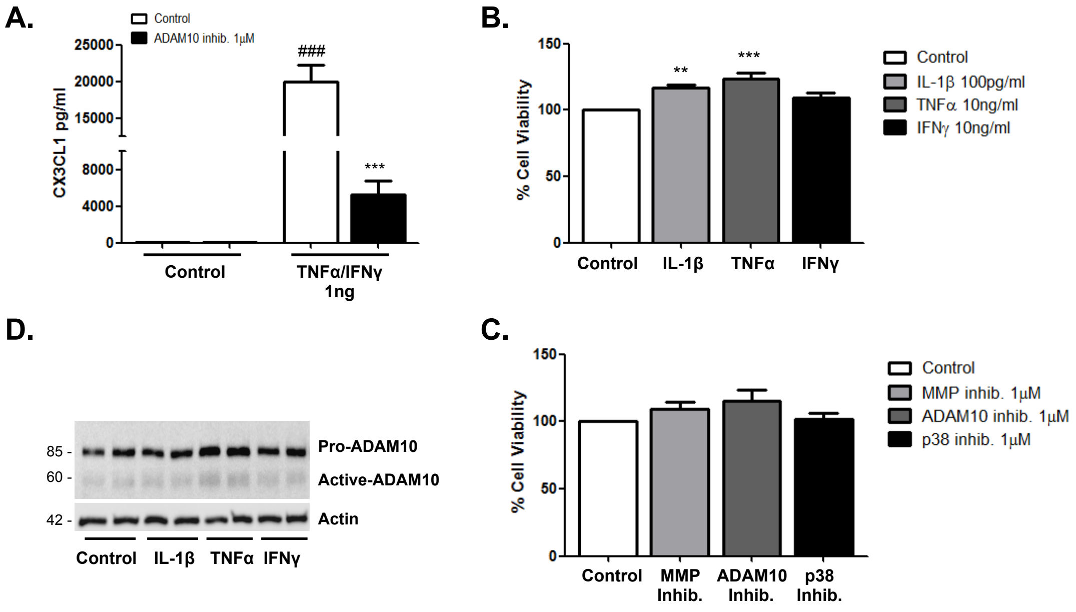

Supplemental Figure 1. (A) Soluble fractalkine levels are attenuated with ADAM10 inhibition following co-stimulation with TNFα and IFNγ. Astrocytes (grown in standard media) were pre-treated with the ADAM10 inhibitor (GI 254023X; 1μM), for 30 min. Cells were then co-stimulated with TNFα (1ng/ml) and IFNγ (1ng/ml) for 18 hr. Quantification of CX3CL1 ELISA revealed a significant decrease in soluble fractalkine when pre-treated with ADAM10 inhibitor. ###p<0.001 compared to own control and ***p<0.001 compared to matched treated group (One way ANOVA and Tukey's post-hoc test). Values expressed as averages +/- SEM; n=4, duplicates. **(B,C)** Changes in levels of sCX3CL1 are not associated with cell death. Astrocytes were treated with **(B)** IL-1β (100pg/ml), TNFα (10ng/ml) and IFNγ (10ng/ml) or **(C)** a pan MMP inhibitor (MMP inhib.) Marimastat (1μM), specific ADAM10 inhibitor (GI 254023X; 1μM) and the p38 inhibitor, VX-702 (1μM) all for 18 hrs. Cell viability was analysed using MTT assay. A significant increase in cell viability is seen with IL-1β and TNFα. **P<0.01 and ***P<0.001; One way ANOVA and Tukey's post hoc test. Values expressed as averages +/- SEM; n=5, triplicates. **(D)** Cytokines do not increase protein levels of the active form of ADAM10. Astrocytes were treated with IL-1β (100pg/ml), TNFα (10ng/ml) and IFNγ (10ng/ml) for 18 hrs before western blotting (representative of 3 independent experiments). In all cases, human astrocytes were serum starved for 3 hr before treatments.
